# The Hospital. Nursing Section

**Published:** 1902-10-11

**Authors:** 


### The Hospital.

### Nursing Section.

Contributions for this Section of "The Hospital" should be addressed to the Editor, "The Hospital" Nursing Section, 28 & 29 Southampton Street, Strand, London, W.C.

No. 837.—Vol. XXXIII. *SATURDAY, OCTOBER* 11, 1902.

### Notes on News from the Nursing World.

### THE KING'S NURSE AT BALMORAL.

The King retains a nurse in his service for the present, and she accompanied the Court to Scotland. His Majesty, who, like Queen Victoria, takes much interest in the home life of the cottagers at Balmoral, heard of the illness of the grandson of an old woman residing on the estate. He accordingly sent his physician to see the young man, but Sir James Reid's report was that the case was hopeless. The next morning the King himself called at the cottage, and having expressed his sympathy, asked to go upstairs to see the patient. He spoke a few cheering words to him, promised that a water-bed should be sent down from the Castle, and told his mother, who was also present, that he would send the nurse who attended him through his serious operation and illness down to the cottage every day, so that she might help to dress her son's wounds and make him comfortable. As may be imagined, the King's kindness and the services of the nurse were vastly appreciated. Notwithstanding all that was done for him, the young man died on Monday.

### THE ROYAL RED CROSS.

We are asked to request any nurses or other ladies now in England who have been awarded the Royal Red Cross, but who have not yet received their insignia, to communicate immediately with the Under-Secretary of State for War, War Office, S.W. The King will hold an Investiture for all Orders on Friday, October 24th.

### TRAINED NURSES FOR SICK PRISONERS.

It is officially intimated that the Home Secretary has given his sanction to the prison authorities obtaining the prompt attendance of trained nurses in the event of the serious illness of prisoners. This is as it should be, and we trust that full advantage will be taken by the prison authorities in all parts of the country for a step which is obviously dictated by motives of humanity.

### NURSING IN SOUTH AFRICA.

The statement made by "A Nursing Sister" in The Hospital last week that people in South Africa are too busy to think of affording the luxury of a trained nurse is not, we must admit, borne out by the remarkable progress of the newly-formed Nurses' Co-operative Society in Johannesburg. In six months it has obtained a membership of seventy-two fully-trained nurses, and the presumption is that there is a demand for their services. We mentioned the scale of fees charged by the co-operation in our issue of June 7th, and they are certainly not such as could be paid by persons with very straitened means. We now learn that the charge for a nurse's service for massage is a guinea per hour. If this, and five guineas a week for an ordinary nurse, can be obtained, the prospect cannot be said to be dark in South Africa; though, of course, there is the danger that the demand for nurses may not keep pace with the increase in the supply.

### CHANGES AT A CAPE COLONY FEVER HOSPITAL.

At the monthly meeting of the Fever Hospital, East London, in September, it was decided after many speeches to accept the resignation of the lady superintendent and the staff of six nurses. Two of the nurses resigned from personal motives but the others decided to leave on account of the statements in the recent report on the fever hospital by Drs. Gregory and Mitchell. A warm discussion took place as to whether Miss McKay's resignation after twelve years' service should be accepted without some recognition of her work. As, however, contributions had materially fallen off, and many charges had been made against the institution, it was decided to accept the resignations, and to make, as far as possible, a thorough change in the whole management. Steps have been taken to fill the vacancies on the staff but it is not expected that many applications will be received, although it was stated that 74 applications were sent in recently at Port Elizabeth Hospital when there were some vacancies. One application for the post of head nurse was favourably considered.

### HOSPITAL NURSES AND DEATH CERTIFICATES.

At an inquest held at Richmond, Surrey, on Thursday, last week, before the Coroner, Dr. Michael Taylor, it transpired that the nurses of the Royal Hospital have been allowed to make out death certificates, and present them to the house surgeon for signature. Mr. Perkins, house surgeon at the Royal Hospital, in the course of his evidence, in admitting the practice, candidly told the Coroner that he had never known it to obtain elsewhere, and he proceeded to add that the irregularity "should not occur again" at the Royal Hospital, Richmond. We strongly advise any hospital nurse who may, in the future, be asked to write out a certificate of death to refer the request to the matron, except in cases in which it is merely a matter of writing at the dictation of the doctor.

### THE NURSES OF HAMPSTEAD HOSPITAL.

Her Royal Highness Princess Christian will lay the foundation stone of the new Hampstead Hospital, at Hampstead Green, on Wednesday,

October 21st, and will receive purses on the occasion towards the building fund. As a purse from the nursing staff, past and present, is to be handed to the Princess, some of the nurses who were trained at the hospital in Parliament Hill, or have been members of the staff, will doubtless be glad to have the opportunity of joining in the gift. Mrs. Ebbetts, the sister superintendent, will be pleased to receive contributions before October 18th.

### UNFOUNDED CHARGES AGAINST A TAMWORTH NURSE.

Specific accusations of unkindness to patients on the part of hospital nurses should always be sifted, but it is only fair that they should not be accepted as gospel unless or until they are proved to be well-founded. The husband of a woman who lately died at her home in Tamworth after her removal from the Cottage Hospital, accused the nurse who attended her with being "too sharp" with her, and with having slapped her, the deceased woman having made statements to that effect. Happily for the nurse, the evidence of other patients in her favour was available at the inquest on the body of the deceased, and another nurse confirmed her assertion that the patient had never complained of ill-treatment. The jury were so well satisfied as to the groundlessness of the charges that they stopped the evidence, and in their verdict exonerated the nurse from any blame.

### A SO-CALLED NURSING HOME AT HOLLOWAY.

The conviction of Mrs. Roach, who on Saturday was fined £25 and £5 5s. costs, or three months' imprisonment, for keeping persons more or less of unsound mind in an unlicensed house at Holloway, is another proof of the folly and risk of attempting to run private lunatic asylums in contravention of the Act of 1890. We entirely agree with Mr. Bodkin who, in stating the case for the Treasury, said that "nothing could be more mischievous than that these so-called nursing homes should be allowed to exist." There is always a danger that they should be made use of by designing people for improper purposes, and they injuriously affect the reputation of genuine nursing homes. In this instance, it was affirmed that Mrs. Roach had "a nurse to assist her in the management" of eleven patients—a number in excess of the accommodation provided—but we notice that the nurse did not give evidence. Mrs. Wainwright, who had been one of the servants "sometimes" employed, testified that a straight jacket was used upon one of two old ladies, and that her hands were fastened by straps to either side of the iron bedstead. There were no charges of improper treatment or cruelty. But one of the Lunacy Commissioners stated that the bedding of the patients in the "nursing home" showed a want of cleanliness, and that the smell was objectionable. The duties of the nurse who "assisted" were, therefore, not adequately discharged.

### NURSES WANTED IN MISSION HOSPITALS.

We understand that qualified nurses are urgently wanted in medical mission hospitals in West Africa. A correspondent, who has been working among the natives for nine years, relates her personal experience. For a short time there was a doctor at the mission station, but, as a rule, she has had neither doctor nor nurse to help her. This means that "she has had to attend to from 10 to 20 patients in a hospital without any of the conveniences of English institutions, to visit patients in their own homes, on dispensing days to prescribe and dispense medicines, dress or superintend the dressings of wounds and sores of from 100 to 150 patients, and to nurse missionaries when sick." She adds that this is a picture of medical mission work generally. If so, there is great room for improvement.

### VISITING NURSES AND FOREIGN LANGUAGES.

It has been found in Philadelphia that linguistic attainments are extremely valuable to visiting nurses. French, German, Italian, and Russian are particularly mentioned, and an amusing account is given in an American periodical of the practical difficulties which a nurse who has to attend a Russian and cannot understand the *patois* of her patient has to encounter. We fear that only a small proportion of nurses can hope to learn Russian; but in London, as on the other side of the Atlantic, a knowledge of even a few words of certain foreign languages is useful. Clever young women who contemplate taking up visiting-nursing after their hospital training might do well to make a point of acquiring at least a smattering of two or three languages in addition to a complete mastery of their own.

### THE ATTENDANTS AT WEST HAM LUNATIC ASYLUM.

In his first annual report on the West Ham Lunatic Asylum, Dr. David Hunter, the medical superintendent, expresses himself very strongly on the question of the attendants. Dr. Hunter says: "In common with most new asylums we have had a number of an unfortunately numerous class who move from asylum to asylum, never remaining long at any place. These people, principally female nurses, have little or no knowledge of their duties, nor do they attempt to acquire such knowledge; they spend their time in grumbling at the necessarily strict rules, and their dietary, and in stirring up a spirit of discontent among the junior nurses. They have not the most elementary idea of discipline, and, above all, to them the patients are a disagreeable and secondary factor, for whom any treatment is good enough. The large majority of the resignations tendered, both male and female, has been indubitably from persons of this class." If so, the West Ham Asylum is better without such persons. But Dr. Hunter's strictures supply a fresh argument in favour of the elevation of the standard for asylum workers; and in this connection the thoughtful letter of "An Asylum Worker" in another column merits careful attention.

### THE RETIREMENT OF MISS LIGERTWOOD.

After being for nearly eighteen years lady superintendent of the Kent Nursing Institution, at West Malling, Miss Helen Ligertwood has retired in consequence of failing health. Miss Ligertwood, who was trained at Guy's Hospital, and was afterwards sister of "Luke" ward, has not only earned the esteem of all with whom she has come in contact, but she has rendered the Institution of which she has so long been the moving spirit most valuable service. We are, therefore, pleased to learn that, at the instance of the Executive Committee, the subscribers have not only decided to set aside as much money as the Institution funds will allow for investment, in order that she may receive the interest during the remainder of her life, but have also started a very requisite supplemental subscription-list, in order to raise a sum to hand to her. Contributions will be received by the honorary treasurer, Colonel Stockley, Nevill Lodge, West Malling, and it is hoped that those of the public who are familiar with the work of the Institution, and have derived benefit from it, will help to swell the amount.

### THE DUAL CONTROL AT CHICHESTER.

The disadvantages of the present system of workhouse nursing were shown the other day at a meeting of the Chichester Board of Guardians. The Board at a previous meeting had decided, without opposition, to increase the salary of the superintendent nurse from £30 to £40. But when a resolution confirming the decision was brought forward one of the guardians protested that "£40 was more than the matron of the workhouse received, and that if they paid that sum to the superintendent nurse they would set her over the head of the matron." The objection was overruled and the superintendent has obtained the increase to which she was clearly entitled. But so long as the untrained matron is supposed to be in any sense in authority over the trained superintendent nurse, incidents of this kind are likely to occur.

### THE NEEDS OF RUSHDEN.

Prominent among modern towns which have made rapid, not to say startling, progress is Rushden, and we are glad to see that it has been determined by the members of the Rushden Nursing Association to put forth steady efforts to secure an income of at least £200 a year. With this amount ensured it is felt that the association can employ two nurses without misgivings, and that two are needed is shown by the fact that in the year recently brought to a conclusion the number of patients visited was 335, and the number of visits paid 9,944. It cannot be long before it will be requisite to consider the engagement of a third nurse. We therefore learn with pleasure that the working classes in the town, who are largely in the majority and who are the only people able to obtain the assistance of the trained nurses, have given fair support to the association in the past, while as to the future, the tone of the speeches at a recent social gathering held at Rushden Hall in order to revive interest in the movement, points to the conclusion that with a little more energy and persistence in collecting, these contributions may be materially augmented.

### COLLAPSE OF A DISTRICT NURSING SOCIETY.

It is not often, we are glad to say, that we have to chronicle the collapse of a district nursing society. At Donaghadee, in Ireland, however, the committee of the District Nursing Association have been obliged to decide upon the discontinuance of their efforts to provide for the needs of the sick poor. The reasons given for arriving at this depressing determination are lack of appreciation of the benefit of a nurse, and the want of adequate support and encouragement by the residents. The organisation will, therefore, cease to exist after the end of next month. It is obvious that district nurses must be paid for their services, and if money sufficient for their salaries cannot be collected, the alternative is to leave the sick poor to seek refuge in the workhouse infirmary, or to go without nursing. But that Donaghadee should have to revert to a state of things which is elsewhere being rapidly altered, is not creditable to the inhabitants who can afford to contribute to the support of the district work.

### DISTRICT NURSES WANTED AT CAISTOR.

The erection of an isolation hospital at Osgodby, near Market Rasen, which will soon be completed, has prompted a suggestion that in association with the hospital there should be provided two or three nurses for the rural district of Caistor. Apart from the practical difficulty that the isolation hospital will be under a different authority from the Caistor Board of Guardians, who have been asked to supply the nurses, there appears to us to be unanswerable force in the objection of the Rev. J. E. Wallis Loft, that the hospital would cease to be isolated if any of the nurses attached to it went about in the country, If, as seems to be the case, at least a couple of nurses for the poor in their own homes are needed in the Caistor district, local energy and spirit should be equal to the task of starting a nursing association which the Caistor Guardians might fitly be invited to assist by means of a liberal grant.

### NURSES FROM SOUTH AFRICA.

The following nurses have lately arrived from South Africa :—On the *Avoca*, Sisters M. E. Farley, A. Mackenzie, R. L. Shappur, M. Fawcett, A. May, L. E. Mitchell, and M. Roche, the two last being invalids; on the *Roslin Castle*, Sister R. C. H. Robertson; on the *Harlech Castle*, Sisters G. M. Dutton and E. M. Chamberlain; on the *Avondale Castle*, Sisters F. L. Gledhill, E. Taylor, H. Freer, and K. Lamont; on the *Carisbrooke Castle*, Sisters E. A. Harmer and E. E. Vincent; and on the *Mohawk*, Sisters C. Meany and A. Myring.

### NURSES' HOME AT NEWTON ABBOT.

The Local Government Board have given their sanction to the plans for the building of a nurses' home in connection with Newton Abbot Workhouse at a cost of £1,423. When the home is finished it will add greatly to the comfort of the staff and remove some, at least, of the grievances of which they have complained in the past.

### NURSES' CO-OPERATION.

As there was not a quorum present at the announced meeting of the Nurses' Co-operation, it was adjourned until Tuesday next at 5.15 The proposed alterations in the rules would, it seems to us, add to the difficulty of securing new members and leave the affairs in the hands of the present authorities, who have not in the past shown themselves mindful of the interests of the nurses.

### Lectures to Nurses on Medical Nursing.

By Eveline A. Cargill, M.D.Brux., L.R.C.P.&S.Ed.

LECTURE VIII.—DISEASES OF THE LUNGS—

*Continued from page* 346.

*Pneumonia* is inflammation of the tissue of the lung or air vesicles, which become filled up with inflammatory products and are rendered solid. It is a very acute disease, accompanied by high fever, and is due to the action of bacteria. The illness comes on very suddenly, generally in strong persons previously in good health, and it often develops after a "chill." The patient becomes extremely ill in a few hours, with severe rigor, a high temperature of 104°—105°, rapid pulse of 100 or more, quick and distressed breathing, the respirations being 40, 60 or even 80 per minute, and the skin hot and dry. The lung becomes rapidly solid, either in part or the whole, and as no air can enter the solid portion there is marked dyspnœa and blueness of the skin. There is usually delirium at the height of the disease.

If one lung only is attacked the chances of recovery are good, but if both lungs are affected (double pneumonia) the case is very serious. The disease lasts six or eight days, and is one of the few serious illnesses that terminates by "crisis." The temperature falls rapidly in a few hours, and the patient as rapidly feels better. There are rarely any complications. Convalescence is short and the patient becomes quite well, the lung being not even weak.

There are few diseases which are so dependent on good nursing as pneumonia; though lasting only a few days the illness is exceedingly severe, and the patient quite helpless and should never be left. Two nurses are desirable, one for day and one for night duty. The great danger is of heart failure, and treatment is directed to relieving the heart, therefore absolute rest must be enjoined; the patient should not be allowed to turn in bed without assistance, and if obliged to sit up because of breathlessness should be carefully raised and the back supported by bed-rest and pillows. The dyspnœa is generally severe, the breathing being very rapid and shallow. Stimulants are necessary in most cases, often in large quantities. When pain is present it is of a sharp stabbing character due to accompanying pleurisy, and may be relieved by hot poultices, otherwise poultices are not advisable. Free ventilation is essential, and the temperature should not be as high as for bronchitis—about 60° is best; the air of the room may he enriched with oxygen, and in pneumonia direct inhalation of oxygen is perhaps more beneficial than in any other disease, and it should be administered whenever signs appear of deficient aeration of the blood. The clothing and bed coverings should be light and loose, a woollen shawl to replace a heavy blanket gives ease to the patient.

Cough is usually present, but is not severe as a rule, and is not to be encouraged, as it is not curative as in bronchitis; neither is there much expectoration, and what sputum there is is tinged with blood or "rusty." A copious liquid "prune juice" sputum sometimes seen in the later stages is of bad omen. Want of sleep is a strain on the nervous system at the height of the illness, and for this tepid sponging may be used, while for continued high temperature cold sponging or cold packs are sometimes ordered, but should never be undertaken by the nurse without very exact instructions. An abundant supply of liquid food is required throughout the illness, about 5 oz. every two hours day and night, or in critical cases food may be required every hour or half-hour. As the crisis approaches the nurse should not leave the bedside, but watch temperature and pulse with the greatest care; sometimes the temperature drops so suddenly that there is severe collapse which must be treated instantly with warmth to the surface (hot blankets and hot bottles), warm drinks, and stimulants, and the patient must not be left for a moment until reaction sets in and the pulse resumes a more natural character. Diarrhœa is a not infrequent complication at the crisis, and a serious one. After the crisis there is great weakness, and constant care is required until convalescence is established.

*Pleurisy*, or inflammation of the pleura or membrane surrounding the lung, generally accompanies pneumonia, but it may also occur independently of any lung disease. The patient should be kept in bed, and allowed to assume the position he finds easiest for the pain, which is characteristic of pleurisy, and is of a severe kind like a knife stab. It is increased on drawing a breath, or coughing, and may be relieved by hot poultices, or by strapping the affected side of the chest, or morphia may be required. The pain is most severe in "dry" pleurisy, and diminishes when fluid is thrown out as in "pleurisy with effusion," but if the fluid is in any quantity the lung is compressed and causes severe dyspnœa. The fluid sometimes has to be drawn off by "tapping," and during the operation the patient is liable to faint owing to the rapid difference of pressure in the thorax, so the nurse should have a stimulant at hand.

Most cases of pleurisy get quite well, but some are liable to relapses, and in some the fluid becomes purulent; the case is then called an "empyema," and requires a considerable surgical operation to drain off the pus. These cases are serious and often take a long time to get well, and then change to sea or mountain air may be necessary, and breathing exercises are useful to expand the damaged lung.

*Phthisis*, or consumption, or pulmonary tuberculosis, is very common in England. It is infectious; and though there is some doubt as to the disease being hereditary, the tendency to contract it is certainly hereditary. It consists of an inflammatory process in which portions of the lung become solid. The inflammation is generally scattered through the lung in minute nodules, which gradually unite and become larger until a large part of the lung may be involved. Some of these nodules break down and leave small holes or "cavities" in their place, the inflammatory *débris* being coughed up and expectorated. As more and more nodules break down the cavities increase in size and may become very large, and if one opens into a blood-vessel serious hæmorrhage will result.

The symptoms of well-established phthisis are but too well known, and when cavities are formed the case is, as a rule, beyond cure; all that can be done is to alleviate suffering and prolong life, and this is best attained in a warm, dry climate.

But in the early stages phthisis is curable, and the best means of cure, as far as our present knowledge goes, is to let the patient constantly inhale *pure* air; it should also be as dry as possible, but not necessarily warm. This is the basis of the open-air treatment now so much employed, the patients in the open-air sanatoria being always either in open verandahs, or in rooms from which the glass windows have been removed. Combined with the pure air there is feeding, to counteract the wasting usual in phthisis, and other treatment as required. The prevention of phthisis is of the utmost importance, and in nursing a case great care should be taken that the disease does not spread. The sputum of phthisical patients is very infectious, and a patient should never expectorate except into a spittoon, and

### LECTURES TO NURSES ON MEDICAL NURSING.—*Continued.*

the sputa should be mixed with carbolic lotion 1 in 20 and then burnt. Handkerchiefs used by the patient should be soaked in a similar lotion before going to the wash, and clothing and bed linen should be scalded.

A patient with phthisis is liable to hæmorrhage at any time, and the nurse must be prepared to deal with it immediately, by making the patient keep perfectly still, lying down, not to speak above a whisper and then only if absolutely necessary. An ice bag may be put on the chest, and ice to the back, but no stimulants may be given. The nurse should try to reassure the patient by a calm confident manner. The hæmorrhage of itself is rarely fatal.

### A Night in a Male Surgical Ward.

BY A NIGHT NURSE.

"Half-past seven, Nurse," said the servant's voice breaking into my dreams, and I tumbled hastily out of bed half awake and groaning inwardly, but became more reconciled to my lot as I dressed; and after a good breakfast with my fellow night nurses went on duty "with a heart for any fate."

When I reached my ward the day nurses went off to supper. Sister gave me the report, not forgetting to apportion me a splint to pad in my spare moments, and went also with a cheery "Good-night," leaving me for the time monarch of all I surveyed. In this provincial hospital of ours, which is perfect of its kind and of which we are very proud, each ward contains 18 beds, so at night only one nurse is needed for a ward. I arranged my night lamp, cut up the boracic lint for the four-hourly fomentations, filled the kettles which had been left half empty by an incorrigible wardmaid, and walked quietly round my domain to see that all was right.

### "Them Flighty Day Nusses."

One or two lucky patients had already forgotten their troubles in sleep, but No. 4, a confirmed grumbler, assured me that his draw-sheet was rucked, and that "them flighty day nusses" had forgotten to fill his hot-water bottle. I found, as I expected, that the rucks existed only in his imagination, and that the bottle was beautifully hot, so I passed on to the next bed. Its occupant, who is eight years old and the pet of the ward, was weeping bitterly with his head under the clothes; "What is the matter, Tommy?" I asked. "My rupture does hurt me!" he wailed in heart-broken tones—he had had herniotomy performed that morning—I reminded him that to-morrow was visiting day, which, with a drink of milk and soda-water, effectually comforted him. A few steps further on I found No. 7, an obstreperous youth with a fractured femur, amusing himself by unfastening his bandages, and was obliged to threaten him with a visit from the house surgeon. No. 11, one of the most cheerful and patient of men, although a bad case of psoas abscess, begged me to leave a feeder full of milk on his locker, so that he need not trouble me during the night; while the dear old man in the end bed wanted to know if I could eat "an egg to my tea," as he had several, and would have been much hurt had I refused it.

### "Prepare the Accident Bed."

At last I sat down to my splint, hoping to get some of it done before the 10 o'clock temperatures and fomentations were due. It is a strange sensation at first to be awake among the sleeping, with the flickering firelight casting strange shadows about the ward, and the stillness only broken by an occasional grunt from a sleeper or the falling of an ember into the grate. At 10 o'clock I took the four-hourly temperatures, etc., and shortly after Night Sister came round to see if anything was wanted. Then I made up the fires and managed to get my splint finished by the time the house surgeon arrived on his last round. "All serene, Nurse?" he asked; "Yes, sir," I replied, and as we were very good friends, I asked him to explain to me the cause and nature of psoas abscess. At 12 o'clock I laid the table in the little kitchen for my solitary meal, and made the tea, but before I had swallowed my first mouthful, a sharp ring made me rush to the telephone, and I heard the ominous words "Prepare the accident bed, Nurse." I had just time to do so when Night Sister and the porter appeared at the ward door with the trolley, and on it a grimy and rather hilarious party. "Pott's fracture, Nurse," said Sister, "it has been put up in the receiving-room." It was obviously many weeks since this gentleman had last been acquainted with soap and water, and it was a work of time to get him clean enough to satisfy Sister; during the process of washing he seemed inclined to weep, and told me in injured tones that the ground suddenly flew up and hit him! However, after a dose of bromide he soon settled down, and I returned to my neglected and now cold supper, which, nevertheless, I swallowed with relish as I was very hungry by that time.

### Preparing Breakfasts.

The next process was to put ready the cups and saucers, and cut the piles of thick bread and butter for breakfast. What appetites surgical men have got! The sky in the east was just beginning to brighten as I finished, and I put my head out of the window to drink in the dewy freshness of the morning air, thinking what beauties the lazy folk in bed were missing; but I could not linger long, as there were enemata and early breakfasts to give to two cases for operation that day, and these had to be done by five o'clock. I got myself a hurried meal of tea and toast, and then put round the washing basins just as it was striking five; it always seems hard to have to wake the patients so early, but it is quite necessary in order to get through the routine of work. Luckily, there were three patients who were allowed to get up and help me with breakfasts, my "orderlies" as I used to call them. After all washings were done and basins cleared away, I set to work to make the tea and fry the bacon, which was ordered for two tubercular patients, while one of my "orderlies," an old man with a poisoned hand, took round the cups and saucers in his sound hand. Another man collected and boiled the eggs, and a boy with a tubercular ankle-joint carried round the bread and butter in his wheel-chair, giving each patient two slices for a first helping. I am always glad to depute the boiling of eggs to one of the men, because it is impossible to please everyone, some wanting theirs hard, others soft, while many are never satisfied. I took round the steaming jug of tea myself. "Rare good tea this morning, Nurse," said one, "it does one a power of good." Poor Tommy had another weep because he was not allowed tea and bread and butter like the rest, and refused to be comforted for a long time.

### Nurses and "Real Ladies."

After breakfast my "orderlies" cleared away and went out on to the balcony for a smoke, while I had one or two dressings to do before making the beds. While behind a screen I heard an amusing argument going on at the other

**A NIGHT IN A MALE SURGICAL WARD—**
*Continued.*

end of the ward between two of the men concerning nurses. No. 1, an old man who had seen better days, said that nurses were real ladies. No. 2 scoffed at the idea, because, he said, ladies would never work so hard. No. 1 replied, "Well, I call them ladies because they behave as such." Whereupon No. 2 burst out laughing and called out to me, "Do you hear that, Nurse? I'm sure you've never been called a lady before!" I did my best to repress a smile, and thanked No. 1 for championing our cause. After this I gave the p. c. medicines, and made the beds on one side of the ward—the other side belongs to the day nurses—Night Sister helping me with one or two serious cases. Then I set to work to clean the sinks and polish taps, and left all clean and neat for the day nurses when they came on duty at 7.30. By 8 o'clock I had to have the report written for Sister, and finally, with a cheery "Good morning, Nurse" from the men, I went off duty feeling that I had earned my outing and my day's rest.

### Fire at Addenbrooke's Hospital, Cambridge.

BY ONE WHO WAS PRESENT.

The serious fire which broke out in the south wing of Addenbrooke's Hospital, Cambridge, resulting in the complete destruction of one ward and in considerable damage to another, was first discovered by one of the wardmaids who happened to be cleaning near. On seeing the flames she gave the alarm, and telephonic communication was promptly made to the fire-brigade in the town.

The fire commenced in the roof, but whether it was due to a defective flue, the fusing of an electric wire, or to carelessness on the part of the workmen, is not known. Fortunately, the ward immediately below was empty, but there were patients in the two smaller wards near, and on the colonnade which is used for the open-air treatment of certain surgical cases; and whilst the porters and workmen were endeavouring to extinguish the flames, the nurses proceeded to convey these patients on their bedsteads to the north wing, which then appeared to be safe. By this time the fire-brigade had made its appearance, and the fire was found to be of greater magnitude than was at first anticipated. The ward on the next floor below was therefore cleared, and the patients conveyed into the garden on mattresses, and as there was no lack of willing helpers, this work was quickly done.

The message then came that the fire was spreading to the north wing, which necessitated the removal of some sixty patients from the first and second floor wards in this wing. These were also conveyed to the gardens, and bedsteads were brought down on which they were placed. The children's ward was likewise emptied. The attention of the helpers was next turned to the furniture, and much of it was removed from the wards and rooms in the vicinity of the fire; also the instrument-cases, glass-tables, etc., from the theatre.

Each sister and her staff of nurses took charge of their own patients, who were by this time lying on bedsteads placed in rows on the lawn: those who were well enough watching the progress of the fire, and the valiant endeavours of the firemen to extinguish it, with much interest.

It was feared that the whole of the south block would be destroyed, and advantage was taken of an offer to put the worse cases into a large empty house just opposite. A staff of two sisters and six nurses were told off to look after the patients, who were carried over on their bedsteads, and who in an incredibly short space of time were enjoying their tea in this temporary hospital. Tea was also served to the patients in the gardens, and everything was done to cheer them and to relieve their minds of anxiety. The nurses, one and all, behaved with admirable coolness and collectedness, and even the newest probationers looked as if a burning roof were an everyday occurrence. There is no doubt that this attitude on the part of the nurses helped to calm the patients and to prevent a panic amongst them. One old "Granny" in the ward below that which was burning remarked: "The nurses know their business and I shall stop here till they tell me to move!"

As soon as the fire was well under, steps were taken to move the patients back into the north wing, and by placing the beds close together, they were all able to be accommodated for the night. Happily, the wards are very large and well-ventilated, so that no harm could come from overcrowding; and by 8 P.M. things had been got fairly straight, and the nurses, tired and wet, were able to go and refresh themselves with hot soup, etc., before retiring to their well-earned rest. The doctors and nurses were indefatigable in their care of the patients, and though there were many serious cases, including abdominal sections and other major operations, amongst those moved, they had the satisfaction of knowing that not a single patient was the worse for the afternoon's disaster.

There were any number of willing workers to assist with carrying patients and in various other ways, and the kindness and sympathy shown by both rich and poor was really quite touching.

### Presentations.

Cancer Hospital, Brompton.—Miss E. A. Stephens, who has resigned her post as theatre sister at the Cancer Hospital, Brompton, to take up that of matron to the Wallasey Cottage Hospital, to which she has just been appointed, was the recipient of the following presents at Brompton on Saturday last:—From the matron, a Russian leather handbag; from the sisters, a pretty china afternoon tea set with oak tray; from the probationers, silver-backed hand-glass, hair brush and comb; and from the maids, silver-mounted pin tray and two clothes brushes.

St. Olave's Union Workhouse, Ladywell.—On Friday last Miss Capes, the assistant matron at St. Olave's Union Workhouse, Ladywell, was presented with a handsome gold watch and illuminated address by the officers of that institution, on leaving to take up the appointment as matron of the St. Olave's Schools at Sutton. The presentation was made by the master, Mr. Norden, who in a few well-chosen words wished her happiness and success in her new appointment. Miss Capes suitably replied, and the remainder of the evening was devoted to vocal music and dancing.

### To Nurses.

**We invite contributions from any of our readers, and shall be glad to pay for "Notes on News from the Nursing World," or for articles describing nursing experiences, or dealing with any nursing question from an original point of view. The minimum payment for contributions is 5s., but we welcome interesting contributions of a column, or a page, in length. It may be added that notices of appointments, entertainments, presentations, and deaths are not paid for, but that we are always glad to receive them. All rejected manuscripts are returned in due course, and all payments for manuscripts used are made as early as possible after the beginning of each quarter.**

### Words of Advice to Nurses.

BY ESTHER H. YOUNG.

### LECTURE II.—UNWRITTEN RULES.

And now we must consider how necessary it is to pay attention to some unwritten Rules regarding certain customs which prevail in Hospital life, points of etiquette, and little matters concerning a very important part of our character—courtesy. We must think not only about "manners" in general, as women who are not ashamed of being women, but more especially about our manners as nurses; towards our patients; towards friends of the patients; towards visitors; and towards one another.

Most of these unwritten rules are just matters of ordinary common sense, or common politeness, so much so, that if in finding fault with a nurse for some breach of the same, one said to her: "Nurse, would you do so and so at home?" she would probably feel shocked, knowing perfectly well that she would not have acted in such a way in her own home, because "mother would not like it" or "father would be angry."

These little things are points which we are almost ashamed to mention, so trivial, that they are seldom spoken of, and so often forgotten, or "taken for granted," that they are put on one side as things which really do not matter!

But let us consider them now, and, as I have already said about printed rules, so I would say about these unwritten ones, "Think, do think!" Nurses are so often heard to say: "I did not know," or "it is not in the rules," or "I was not told that!" etc., that one is inclined to ask them: "Nurse, is it on your printed rules that both stockings must be put on every day?"

I think you will be astonished at my first "unwritten rule," and I hope it is almost unnecessary to mention it, but perhaps it may be of use to some of you! In most of the printed rules you find something like this:—"Medicines and stimulants which must not be taken except when prescribed by the doctor, must be given into the charge of the assistant matron, or a superior officer." This practically forbids stimulants being kept in your bedrooms, but the words "Smoking is not allowed," are not often added. But surely this is never permitted, and you must know that it is against the wishes of those in authority over you. Smoking is not a womanly habit, and certainly it is most unfitting for any nurse.

Again, in your printed rules you will probably find a clause forbidding you to leave any food about in the dormitories. Provision is usually made to enable you to get tea near your sleeping rooms, or in your sitting rooms, but I am sure that the less food you take in your dormitories and the fewer supper parties you have, the better for you.

Plenty of good food is provided for you in your dining hall, and those extra meals are so often bad for your health, producing indigestion, headache, and so on. They also lead to breaking rules, and certainly prevent you from forming good habits.

For instance, you cook the food on a gas stove, the smell is objectionable to others; or you have a supper party, the noise is a nuisance to those in other cubicles; and is it quite over by 10 o'clock, so that each nurse returns to her right division of the dormitories by that time? Or, if you all belong to the same division in which the party takes place, is it all over, and everything washed up and put away, are all of you in bed, and quite ready to go to sleep by 10.45 P.M., when "lights are put out, and silence is to be observed"? Is it not too often the case that long after 10 o'clock, one or two of you (or more!) have to go to another set of cubicles, thereby breaking one of the Hospital rules? And does it not often happen that just at the last minute, when Sister comes round to put the lights out, cups and saucers are put away dirty until the morning, each nurse hurries to her cubicle, and scrambles into bed, with duties, both spiritual and bodily, left undone?

When morning comes, you are very tired and get up late. Cups and saucers must be washed, and you have little time for this, so you arrive in the dining hall after grace has been said, and that dreadful "late" mark has to be placed against your name. Surely, both morning and evening, many valuable minutes have been wasted, and important duties neglected! At night, in consequence of that supper party, and the necessary hurry in getting into bed, there was no time to give thanks to God for His blessings during the day, nor to ask His forgiveness for failures, nor to commend to His Fatherly care your loved ones, relatives and friends far away. No time, in fact, to say your prayers at all, or perhaps just time to say a few words without much thought or care. There is the same tale in the morning, no time for prayers, or at least no time before breakfast, possibly it was put off to be managed somehow later on in the day, "got through" just as a form, lacking thought or reverence, or realisation of the presence of God.

Days begun and ended in this way, with such work as nursing in our hands, are (we know it very well!) unsatisfactory, and the sooner we discover this fact for ourselves, the better for us! We have not only upset our own interior life, but also that of others. Nurses are not school girls (or boys!) and they really do not need "tuck" in their bedrooms! So I ask you to consider one another's real welfare in this matter. Consider the need of your souls before the luxuries of your bodies.

In many hospitals there is some such rule as this: "While on duty you may not receive visitors." And there is an important "unwritten rule" which common sense points out as necessarily accompanying it, for it is evident that visitors cannot be admitted into the wards for the purpose of calling on the nurses and probationers. And yet, from time to time, one finds that nurses are surprised at this arrangement, and their friends feel aggrieved! If there is no home with a waiting room for visitors, you will do well to make arrangements with your friends to fetch you from your hospital.

If you wish to take friends over the hospital you should ask the matron's permission. And when you bring them into any ward always show politeness to the sister, or in her absence the head or staff nurse, by asking if you may go round the ward with your friends.

There is another custom in all hospitals which it may be well to explain, I mean the habit of standing in the presence of your superiors. At one time nurses had to stand very much more than was good for them, or really necessary, and it is well that in this respect, as in so many others, an improvement has been made.

Unless you are told to sit, stand while the doctors make their rounds, when the sister comes up the ward to speak to you, when the matron is going round, and also when visitors are being taken over the hospital by anyone.

Again it is the custom to use certain modes of address in speaking to particular individuals, and the reason for this is obvious. In answering questions we cannot reiterate "Miss," "Mrs.," "Mr.," or "Dr. So-and-so,"—neither is it quite polite to say merely "Yes" or "No," therefore for many years it has been the custom for nurses to address each as "Nurse," those next in authority in the wards or in the home as "Sister," and for all to address the matron as

### WORDS OF ADVICE TO NURSES—*Continued.*

"Matron." In speaking to any of the visiting staff we say "Sir." All these little matters are points of etiquette, not personal, but showing respect to the various offices held by the different individuals.

Probationers should also show respect to head nurses, and juniors to seniors, but the new probationer of to-day is sometimes lacking in that rather wholesome awe of everybody, which is really to her advantage.

In the wards the probationers should be obedient to "nurse," and be ready to be taught by her. In the life outside the wards, though all should feel a sense of comradeship and fellowship, yet probationers should be careful always to make way for "nurse," let her go first, open doors for her, and be ready to do little things for her, just in the same way that the nurses in their turn show respect to their special "Sister" and to all the sisters.

Now let us pass on from the consideration of these written and unwritten rules, and turn our thoughts to the subject of Courtesy.

We, in hospitals, come in contact with humanity in very varied forms, some very horrible, some approaching the divine.

The very word "Humanitas" in Latin means the manners befitting one human being towards another, and therefore, "good breeding, gentleness, kindness, politeness." So let us think about Courtesy, first of all in the wider sense, and then more in detail.

Courtesy is such a very necessary virtue for all people, and especially for all women, members of the "gentler sex," but it is not one of the easiest habits to acquire and keep, though essential. To be constant and unwearying in all relations, at all times, towards all people, is a rare grace, but it is a most important part of a good woman's character. There is a singular power and distinction in those few lives in which we feel sure its presence is unfailing. I can recall a striking instance of one under whom I worked for some time, who for fourteen years was matron of one of our well-known provincial hospitals and whom to know was to love.

Perhaps we are apt to look upon this grace more as an attractive ornament—something that we cannot hope to attain to, and only to be admired by us in others—more than as an intrinsic part of that perfection at which we have already distinctly made up our minds to aim!

That thought reminds me of those lines of Kingsley's which were said to me some years ago by someone who now lives in one of our largest hospitals, and can influence for good so very many new probationers, though she is not actually at work amongst them. I was then a new "pro.," wondering if I should ever become a good nurse, and she quoted:

"Be good, sweet maid, and let who will be clever,
Do noble things, not dream them all day long:
And so make Life, Death, and that vast For Ever
one grand, sweet song."

But it is so difficult to be always good, is'nt it, and, like a baby patient whom I once knew—we find that we want to be cross, not polite, "'cos me do!" Little Jabez was ill, and he was only three years old. He was always very pleased to see me, until one day when he was cross, and when asked why, he just said: "'Cos I is!"

But we are more than three years old, and we are not ill, so we must take care to cultivate our "manner," which is the outward expression of this virtue of courtesy, though many of us seem to think of it as a matter of very little consequence! So and so is sharp, "off-hand," and others say: "Oh, it is only her manner, she can't help it!" It should not be possible to say this about any nurse, often, this offending "manner" is caused by sheer thoughtlessness, or want of consideration for other people's feelings.

Think for a moment, or rather for a few seconds, and that cutting remark will not be uttered, that bored expression will give place to a smile, those shoulders will only begin to go up! Don't be pleased or amused if anyone says to you: "You take heads off with such a very clean cut," etc., etc.

Besides this habit of thinking before you speak, which is a wonderful check on impatience, there is another which is a great help towards the cultivation of real politeness in manner and speech, I mean speaking slowly. Perhaps you have noticed that those people who speak slowly are rarely so impatient as those who talk quickly. To one accustomed to answering at once, and talking quickly, it is a great effort to measure each word, to pause before speaking, to "put the brake on" that unruly member, the tongue, but I can assure you that the effort is well worth making!

(*To be continued.*)

### Everybody's Opinion.

**[Correspondence on all subjects is invited, but we cannot in any way be responsible for the opinions expressed by our correspondents. No communication can be entertained if the name and address of the correspondent are not given as a guarantee of good faith, but not necessarily for publication. All correspondents should write on one side of the paper only.]**

### VILLAGE NURSES IN CUMBERLAND.

"The Countess of Lonsdale, President of the Cumberland Nursing Association," writes from Lowther, Penrith: In your number of September 27th there appeared, under the heading "Cottage Nurses in Cumberland," a notice of a bazaar lately held at Penrith in aid of the funds of the Cumberland Nursing Association, in which, after describing the number of nurses and their training, the following lines conclude the paragraph:—"We cannot help feeling, however, that 'the towns, villages, and scattered districts of Cumberland' in which they are engaged, must remain at a disadvantage compared with other counties where fully-trained district nurses are employed." As this statement appears to me misleading and hardly fair to Cumberland nurses, I shall be grateful if you will be good enough to insert in your paper my explanation and some facts concerning the nursing in the county. In the first place, I cannot admit that Cumberland employs fewer fully-trained district nurses than other counties—on the contrary, I think it stands high in this respect. In the towns and mining centres, Queen's nurses are employed: six of these are working in districts affiliated to the county association—at Maryport, Cockermouth, Millom, Cleator (two nurses), and Longtown—whilst there are several in the towns of Carlisle, Whitehaven, and Penrith, independent of the Cumberland Nursing Association. It is the great wish of the Cumberland Nursing Association that a fully-trained nurse should be employed wherever it is possible to support one, but in the scattered village districts of the county, sufficient funds cannot be collected to pay the heavy cost of such a nurse; therefore, one at a less cost must be provided. The association trains young women, who are called "village" (not cottage) nurses—and so recognised by Queen Victoria's Jubilee Institute—who, after their training at Plaistow, return to Cumberland, being bound to do district nursing under the association for a period of three years at least. It should be noted that these village nurses are in most cases Cumberland women understanding the ways of the people they are to work amongst in future, and are carefully selected by the superintendent and committee of the Cumberland Nursing Association before they are sent to Plaistow for training. Without wishing in the slightest degree to make any comparison between nurses, I can only give my opinion after five years' experience in Cumberland—that the "village" nurse who has nine months' training in district nursing and holds a first-class midwifery certificate is equally as efficient for the particular work required of her as her more fully-trained sister. Moreover, it is now well known and acknowledged that the maternity nurses trained specially for district work are much more successful in country villages than the *hospital* nurse who has had only a *smattering* of district nursing. Since writing the above a copy of The Hospital of October 4th has been put into my hand in which a letter appears written by "A. B." ("who is not a nurse and resides in Cumberland") cordially agreeing with the statement in your issue of September 27th about fully-trained nurses, to which I have already referred. But "A. B." goes on to say "in one instance which has come under my notice some members of a branch of the Cumberland Nursing Association have done their level best to try to drive an experienced nurse away for the sake of keeping a cottage nurse whom three-quarters of the inhabitants will not employ or subscribe to, having by their own experience discovered the advantages of a fully-trained district nurse." This is an assertion against "some members of a branch of the Cumberland Nursing Association" which needs substantiating, and which, unless proved, I must absolutely deny. May I ask "A. B." to make his or her complaint to the proper source. It is not fair on the Cumberland Nursing Association to have charges brought against the association and its nurses by those who have not even the courage to sign their communications to your valuable paper.

### NURSING IN THE ISLAND OF JERSEY.

"An English Nurse" writes: In reference to your article in this week's Hospital on "Nursing in the Island of Jersey," will you allow me to say a few words about private nursing in the island? I have been working in St. Helier for several years and know of at least ten other private nurses here, while there may be others whom I do not know. The people here, as a rule, prefer untrained nurses who will do the cooking and at least part of the housework as well as nursing the patient. Servants are very difficult to obtain and are often unsatisfactory, so, when possible, people do without them. It is not unusual to have an interval of one or two months between each case. The usual fee charged for a medical or maternity case is one guinea a week, two guineas for an operation case, and 30s. for fever. One or two of the principal doctors have their own nurses and employ no others. The French sisters you mention are usually employed by the French population only, as I believe the majority of them speak nothing but their own language. I remember being sent for by a doctor some time ago to assist him during a confinement. One of the French sisters was in attendance, and both she, the patient, and the other members of the family were unable to understand or speak one word of English. During the time I have been here I have known several nurses leave the island because they were unable to obtain sufficient employment, so it would, I am afraid, only mean disappointment for any others who might come.

### THE FUTURE OF THE ASYLUM NURSE.

"An Asylum Worker" writes: After the very able and sympathetic article which appeared in your columns on September 20th, entitled "The Better Nursing of the Insane," it seems almost presumptuous for one so humble as an asylum nurse to attempt anything on the same subject, but if it will not occupy too much valuable space I should be glad to try and place before the "nursing world" a very real difficulty regarding the practical side of mental nursing. One acquainted with the nursing of the insane may well be astonished at the contempt in which this work is held, amongst people in general, and the ignorance they display of mental illness. To many persons an asylum is vaguely a large building in some remote spot something resembling a prison, full of raving maniacs who are guarded by a grim stoic-like female, whose nature, though feminine, can never be credited with anything approaching womanliness. Few seem to realise that the "keeper" of the old school belongs to a time long passed, and the present effort is to induce women of education to enter the ranks, become acquainted with the various phases of mental disease, acquire tact and discretion in dealing with the different characters, and above all to cultivate a spirit of quiet sympathy, more necessary for winning the confidence of those afflicted than a flow of light merriment, into the spirit of which those bowed down with misery feel they cannot enter. It may be said that if this work has been effectually discharged by women of a humble station it is not fair that they should be ousted in favour of their more fortunate sisters, but in this, as in all advances, the few must suffer for the good of the many, and of those entering to gain their experience it is not necessarily the well-educated and accomplished woman only who will develop into the good nurse. A certain amount of refinement is desirable, but in these days of education surely the necessary requirements are within reach of the most humble, and there is an equal chance for the lady and for the "working woman." Supposing then that a body of suitable women enter an asylum, acquire their knowledge and complete their term of training satisfactorily, may I ask what is to be the future of these nurses? In general hospitals the most efficient nurses gradually rise to higher positions as "sister" of a ward and eventually matron of a hospital. One would naturally expect an equal opportunity in this speciality, but is it not the increasing tendency in asylums to almost exclusively fill the higher posts with fully-trained hospital nurses, in preference to the woman who has trained in the institution, and who knows her work? How often do we not read that "Fully-trained hospital nurses are required to take charge of an asylum ward where a knowledge of mental nursing is not necessary?" Is mental nursing then so simple that it requires no learning, or is the sister of this especial ward not responsible for the welfare of her patients? and, if so, why are women required to train before they can be considered competent mental nurses? Above all, how is the woman, inexperienced in the branch of work she undertakes, to understand the requirements of those under her care, and how can she teach her probationers what she does not know herself? A fully-trained hospital nurse one day remarked to me with surprise that the superintendent of a large asylum had asked her to undertake the charge of a ward in his institution, and when she assured him that she had no experience whatever of mental nursing, he replied, "Oh, you will soon pick that up." She remarked, "One would never think of placing a trained mental nurse at the head of a surgical ward and telling her that her knowledge of mental nursing was sufficient to guide the ward till she should have 'picked up' from her probationers a sufficient proficiency in surgical work." Nor do we ever read of any matron requiring a mental nurse for the head of a hospital ward where a knowledge of sick nursing is not necessary. On the other hand, we are asked, "Are the insane never sick?" and "is not some knowledge of sick nursing required from the asylum nurse?" Those acquainted with the care of the insane know that, though illness is comparatively rare in asylums, an attack of insanity is almost always accompanied by some physical ailment, and more especially is it necessary that the nurse should be observant of the condition of her patient, because the insane are apt to complain without cause, or equally to hide any sign of physical discomfort. For those who desire to undertake the care of the insane, a knowledge of sick nursing is therefore desirable—preferably a full term of training—but as sick nursing is a vocation practically open to all classes of educated women, who could be induced to serve a term of training in an asylum first, if at the end of that time they must start afresh in a general hospital in order to acquire the qualification necessary to enable them to climb to the head of the tree. On the other hand, by qualifying in the latter branch first, they may at once secure one of the higher berths in asylum work. And granted that a lesser term of sick training is sufficient, how many nurses are able to save from their salary a sufficient sum to enable them to secure by payment this extra training for themselves? The only opening then for the mental nurse is to linger on in the institution which has trained her or else resort to the more lucrative branch of private nursing. But surely few who really care for their work will be content indefinitely with the former, while the latter course tends to empty the asylums of their most suitable workers; and the greatest difficulty is that the doors of advance being closed, the most promising women will necessarily draft off into other, and more attractive, branches of nursing. Unless a means is provided for enabling the asylum nurse to become fully proficient, will the superintendents continue to have a choice of the best material for training, and if not, how is the tone of asylum nursing to be raised to a higher level?

### A NEW WRINKLE IN ADMINISTERING OIL OR NUTRITIVE ENEMATA.

"Sister Helen" writes from Visalia Sanitorium, California, U.S.A.: Not being satisfied with the method of administering oil, or nutriment by enema, I tried an experiment, which has turned out very satisfactory. Used with a fountain syringe, I had made a large glass-bulb tubed off at both ends to hold about a pint and a half of oil, &c. I cut off ten inches from the nozzle end of syringe tubing, fastened one end of each severed piece on each end of the tube ends of glass bulb; which of course left the glass bulb ten inches from nozzle end and about four feet from the top. When the bowels had been cleaned and the rectal tube inserted the necessary length, I poured into the top the requisite amount of oil, having ready a pitcher of the soapsuds to pour into the bag, as the oil in the bulb disappeared from view; in this way I was certain that no oil was left outside, and also that it went direct to the spot. It has also this advantage, that if one wishes no oil to enter the rubber bag (which every nurse knows is injurious to the rubber, and also quite a good deal of work to clean, if bag is needed for a douche), one can fill the glass bulb, then join the upper tubing to it. To fill it thus I have invented a rubber funnel (a very home-made article it would look to most, but some day I will have one made properly). The tube end of this fits into the glass-tube end to fill the latter with oil—it is also very useful to fill medicine bottles with narrow necks with specimens of urine—insuring no waste where specimens are scanty in quantity, the whole amount entering the bottle.

### NURSES' READING SOCIETY.

"L. M. Moberly" writes from Belsize Cottage, Belsize Lane, N.W.: The following are the prize winners in this year's examinations:—Miss J. Till and Miss Milligan, 400 marks each; Miss O. Wilson, 385 marks; Miss Marsh, 355 marks. The two bracketed together have made 100, the maximum number of marks, every quarter. The sum of £1 has been distributed amongst the four nurses above mentioned. Owing to the falling off in the number of members and to my own increased literary work, I am now obliged to bring the society to an end, but I hope that all those members who have co-operated with me so heartily and cordially will write to me whenever they can do so, if they think I can help them by recommending books, or in any other way. Those members who have been faithful to the society have written me such warm letters of gratitude that I should like to thank them collectively for their help and support during the years of the society's existence. I can only hope that the society itself while it lasted was some help to those anxious to keep up their theoretical, as well as their practical, work.

### A HARD CASE.

"Lillian Davidson" writes from Church House, Buxton Street, E.: Will the following nurses please accept the most grateful thanks of the District Nurse they have so kindly helped:—"Private Nurse," Weymouth; "Anon," Middlesbrough; "Enteric Ward," Halesowen Hospital; "Anon," Nurses' Home, Frederick Street, Belfast; "District Nurse," Kendal; "Sympathiser," Bradford; "J. E. W.," Kilburn; "A Fellow Nurse," Kingston-on-Thames; "E.," Lichfield; "Sympathiser," Birkenhead; and "Anonymous," Birmingham.

### TRAVEL NOTES AND QUERIES.

France to Acquire a Good Accent (Nurse S.).—Thanks for your letter on the above, if you will kindly give me some particulars as to terms you will increase my obligation. An address without terms is but little good, as the question of expense is a burning one to all. I think I have heard from you before—is it not so?

### Appointments.

Bagthorpe Infirmary, Nottingham. — Miss Annie Elizabeth Cawood has been appointed sister. She was trained at the Fever Hospital, Blackpool, for one year, and at Middlesbrough Union Infirmary for three years. She has since been charge nurse at the latter institution. Miss Cawood holds the L.O.S. certificate.

Barnet Union.—Miss Dora Lascelles and Miss Marian Summers have been appointed assistant nurses. Miss Lascelles was trained by the Meath Workhouse Nursing Association at the Invalid Asylum, Stoke Newington. Miss Summers was trained by the Meath Workhouse Nursing Association at St. Peter's Home, Kilburn.

Birkenhead Union Infirmary.—Miss Elizabeth Varty has been appointed temporary charge nurse. She was trained at Birmingham Union Infirmary, and has since been charge nurse at Bolton Union Infirmary, Kendal Union, and Fulwood Union, Preston.

Bromyard Cottage Hospital.—Miss Lillian Smith has been appointed nurse-matron. She was trained at Salford Royal Hospital, Manchester, where she has since been sister. She has also done private nursing, and as a member of the Army Service Reserve was for two years nursing the sick and wounded in South Africa.

Bucknall Hospital, Stoke-upon-Trent. — Miss Georgina Keith Gillow has been appointed charge nurse. She was trained at Leith Public Health Hospital, East Pillon, Edinburgh, and has acted as senior assistant nurse for four years.

Bucklow Union, Knutsford.—Miss Mary J. Boyce and Miss Ellen Fellows have been appointed assistant nurses. Miss Boyce was trained by the Meath Workhouse Nursing Association at the Children's Hospital, Temple Street, Dublin. Miss Fellows was trained by the Meath Workhouse Nursing Association at St. Peter's Home, Kilburn.

Chelsea Hospital for Women.—Miss Annie Tate has been appointed sister. She was trained at St. Mary's Hospital, Paddington, and has since been staff nurse in the same institution, and sister for two years and a half of the women's ward at Southwark Union Infirmary, Dulwich.

Colchester Hospital.—Miss Annie Dunn and Miss L. Geddys Buck have been appointed sisters. Miss Dunn was trained for three years at the Royal Infirmary, Hull, where she has since done temporary duty as night sister and casualty sister. Miss Buck was trained for three years at the Royal Portsmouth Hospital. She has since done nursing in a surgical home in London for nine months, and been sister at Lewisham Workhouse Infirmary.

Durham County Hospital.—Miss Margaret Whitlock has been appointed matron. She was trained at Durham County Hospital, where she has since been sister-in-charge.

Epsom Workhouse Infirmary.—Miss Rose Beer has been appointed charge nurse. She was trained at the Union Hospital, Cardiff, and holds the L.O.S. certificate.

Royal Albert Edward Infirmary, Wigan. — Miss A. M. Holleley has been appointed theatre sister. She was trained for four years at St. Thomas's Hospital, London, and has since been sister at the English Hospital, Cairo; holiday sister at the Children's Hospital, Shadwell; charge nurse and night sister (*pro tem.*) at the Brook Fever Hospital, London.

Torbay Hospital, Torquay.—Miss Ethel S. Fortescue has been appointed matron. She was trained for three years at the Lincoln County Hospital, where she was afterwards staff nurse, and at the City of London Lying-in Hospital. She has been matron at the Convalescent Hospital, Brixham. She holds the L.O.S. certificate.

Union Infirmary, Limavady, Derry.— Miss Emily Callwell has been appointed superintendent nurse. She was trained at the East End Children's Hospital, London, the Royal Victoria Hospital, Belfast, and the British Lying-in Hospital, Endell Street, London.

### Echoes from the Outside World.

### The King's Drive through South London.

The King entertained his tenantry and servants at a supper and ball at the Castle on Monday before leaving Balmoral for his visit to North Berwick on his way to London. His Majesty has approved of the route of the Royal Procession for Saturday the 25th inst. It will be identical with that originally selected for June 27th, viz., the Mall, Marlborough Gate, Pall Mall, the north side of Trafalgar Square, the Strand, Fleet Street, Ludgate Hill, St. Paul's Churchyard, Cannon Street, Queen Victoria Street, the Mansion House, Prince's Street, Gresham Street, to the Guildhall, returning by King William Street, London Bridge, Borough High Street, Borough Road, St. George's Circus, Westminster Bridge Road, Westminster Bridge, Bridge Street, round Parliament Square, Parliament Street, Whitehall, the Horse Guards, and the Mall to Buckingham Palace. The Thanksgiving Service at St. Paul's Cathedral on the following day will be of a private character, similar to that which took place upon Peace Sunday. Their Majesties will drive along Victoria Street, the Embankment and Ludgate Hill and return *via* Holborn, Oxford Street, Hyde Park and Constitution Hill. There will be no troops to line the streets but on the 25th it is expected that about 50,000 soldiers will be brought into London. It is not yet officially stated whether the Guards and some of the *Terrible* men will take part in the procession, but it is evident that the pageant will be made as imposing as possible.

### Royalty at Home and Abroad.

Queen Alexandra on Saturday attended a grand review of troops at Copenhagen, in company with her sister, the Empress Dowager of Russia. Their father, King Christian—who, notwithstanding his 84 years, still rides like a young man—was on horseback. All the Royal Family attended divine service next day, and had a dinner party in the evening. The Crown Prince of Greece, who is an enthusiastic motorist, last week had an accident whilst driving, the result of the unsatisfactory state of the roads between Athens and the Summer Palace at Tatoi. The fall of the automobile was fortunately arrested by some pine trees, or it might have pitched down a deep ravine which opens below the road. Three doctors were speedily in attendance, the cuts round the eye and the lip were successfully stitched, and it is hoped that no complications or fever will arise. The King of Portugal is expected to pay a visit shortly to King Edward in London. It has been stated that the German Kaiser will come to meet King Charles, but this is not regarded as probable, for the King's visit is expressly intended to be of a personal character.

### The Coal Question in America.

The coal strike in America still goes on, and President Roosevelt—who is now almost well again—has not as yet come to a decision as to what can be done in the matter. One hundred and forty-seven thousand miners are out on strike, coal in some parts of New York is as high as 75s. a ton, and in some of the hospitals there is not a sufficient supply to last a fortnight. The coal companies affirm that if they are afforded adequate military protection they can employ non-union miners and get out plenty of coal, but the strike leaders maintain that the miners on strike are skilled workmen, and that non-union labour cannot safely or profitably do the work. Mr. Roosevelt is opposed to sending Federal troops to guard the coal mines, and thinks that Pennsylvania authorities should deal with the situation themselves. The Pennsylvania Militia numbers 1,000,000 men and Governor Stone declares he will, if necessary, call them all out. Meanwhile, the poor of New York are likely to suffer far less than the lower classes in other towns, as Mr. Pierpont Morgan has arranged to purchase 50,000 tons of Welsh anthracite for distribution amongst the poor and the public institutions of New York. The ships of the new Shipping Combine will bring over the coal.

### Melodrama at His Majesty's Theatre.

Shakespearean revivals have given place, at His Majesty's Theatre, to a version of Mr. Hall Caine's novel, "The Eternal City," dramatised by himself. In the play the political business, which is a primary feature of the novel, is not introduced, but in other respects the romance is followed as closely as circumstances will permit. The drama is divided into five acts and eight scenes, all of which are splendidly mounted, several being very masterly in design. The dresses, too, are magnificent. The plot is not very clear to those who have not perused the novel, though the motive, a woman's desire for revenge turned to love, is sufficiently apparent. The first scene, which represents the loggia of Baron Bonelli's palace, shows a party of noble Romans assembled to see the Pope going in State to St. Peter's. Amongst these is the beautiful Roma, Bonelli's ward and mistress. A revolutionary young man, David Rossi, insults Roma, who undertakes to punish him herself. When she is interviewing Rossi Roma finds that she used to play with him in her childhood, succeeds in gaining his affections, and herself falls in love with him. Then Rossi gets mixed up with an insurrection, and flies the country. Roma is counselled by the Pope to betray the whereabouts of her lover to the police, and she does so, trusting to the Pope's promise that David's life will be spared. Afterwards Bonelli tells her that the promise will have to be broken, but David escapes from his captors, comes to Roma's studio, shoots Bonelli, and forces his way into the Pope's private apartments and claims sanctuary. The Pope takes the opportunity to reveal himself as David's long-lost father. Finally a revolution takes place, Rossi becomes Prime Minister, and is happily united to Roma. The part of Roma is played by Miss Constance Collier, who looks exceedingly handsome and plays with much passion. Mr. Robert Taber is a romantic Rossi, Mr. Brandon Thomas a dignified Pope, while Mr. Tree as an Italian Jew is cleverly "made up" and impressively wicked. Mr. Lionel Brough, as Rossi's faithful friend Rocco, scored a distinct triumph in a small part.

### The Photographic Exhibition.

The annual exhibition of the Royal Photographic Society is now open, at the New Gallery, Regent Street. The pictorial section, with nearly three hundred works by amateurs and professionals, is the most interesting to the casual observer, but the numerous scientific applications of photography, exceeding a hundred in number, are the most important feature. Dr. Clowes sends a fine series of photo-micrographs of bacteria, made in connection with the bacterial treatment of sewage of the London County Council, some of which are shown magnified as much as three thousand diameters. There are several photographs by the Rev. J. M. Bacon, taken from a balloon, while a large carbon-print of a lion's head, entitled "Sultan," by Mr. W. L. Wasteli, is a practical proof that a striking and most successful result may be secured by a single snapshot at the Zoological Gardens. The contribution by Mr. Douglas English of a little platinotype print taken from life, which shows two mice fighting in much the same attitudes as human wrestlers, is in the pictorial section; but the rest of his clever series of animal life are more appropriately placed in the balcony.

### For Reading to the Sick.

HE HEALETH THE BROKEN IN HEART. HE TELLETH THE NUMBER OF THE STARS.—*Ps.* cxlvii., 3, 4.

Teach me your mood, O patient stars!
Who climb each night the ancient sky,
Leaving on space no shade, no scars,
No trace of age, no fear to die.
*R. W. Emerson.*

I looked up to the heavens once more, and the quietness of the stars seemed to reproach me. "We are safe up here," they seemed to say; "we shine, fearless and confident, for the God who gave the primrose its rough leaves to hide it from the blast of uneven spring, hangs us in the awful hollows of space. We cannot fall out of His safety. Lift up your eyes on high, and behold! Who hath created these things—that bringeth out their host by number? He calleth them all by names. By the greatness of His might, for that He is strong in power, not one faileth. Why sayest thou, O Jacob! and speakest, O Israel! my way is hid from the Lord, and my judgment is passed over from my God?" —*G. MacDonald.*

I love to think that God appoints
My portion day by day;
Events of life are in His hand,
And I would only say,
Appoint them in Thine own good time,
And in Thine own best way.
*A. L. Waring.*

"There shall no evil befall thee, neither shall any plague come nigh thy dwelling," is a promise to the fullest extent verified in the case of all "who dwell in the secret place of the Most High." To them sorrows are not "evils," sicknesses are not "plagues;" the shadow of the Almighty extending far around those who abide under it, alters the character of all things which come within its influence.
*Anon.*

A root set in the finest soil, in the best climate, and blessed with all that sun and air and rain can do for it, is not in so sure a way of its growth to perfection, as every man may be, whose spirit aspires after all that which God is ready and infinitely desirous to give him. For the sun meets not the springing bud that stretches towards him with half that certainty, as God, the source of all good, communicates Himself to the soul that longs to partake of Him.—*Wm. Law.*

All my life I still have found,
And I will forget it never;
Every sorrow hath its bound,
And no cross endures for ever.
All things else have but their day,
God's love only lasts for aye.
*P. Gerhardt.*

We never have more than we can bear. The present hour we are always able to endure. As our day, so is our strength. If the trials of many years were gathered into one, they would overwhelm us; therefore, in pity to our little strength, He sends first one, then another. We do not enough look at our trials in this continuous and successive view. Each one is sent to teach us something, and altogether they have a lesson which is beyond the power of any to teach alone.
*H. E. Manning.*

### Notes and Queries.

**The Editor is always willing to answer in this column, without any fee, all reasonable questions, as soon as possible.**

**But the following rules must be carefully observed:—**

**1. Every communication must be accompanied by the name and address of the writer.**

**2. The question must always bear upon nursing, directly or indirectly.**

**If an answer is required by letter a fee of half-a-crown must be enclosed with the note containing the inquiry, and we cannot undertake to forward letters addressed to correspondents making inquiries. It is therefore requested that our readers will not enclose either a stamp or a stamped envelope.**

*Training.*

(6) Miss F. A. Davies, the Sanatorium, Crooksbury Ridges, Farnham, Surrey, and the matron of the Dowsing Institution, 28 York Place, Baker Street, W., would like to hear from "R. R."

*Fees.*

(7) I have been for six and a half years in a nursing home in Scotland, and I have been in the City of London Maternity Hospital for a course of monthly nursing; I have also been a district nurse for three and a half years, and now I am going back to Scotland to take up private work. As I want to have some cards printed, would you kindly tell me what fees I might charge?—*Little Nurse.*

You must arrange the scale of fees in accordance with those charged in the vicinity in which you set up, having reference also to the class of patients you are likely to obtain. Well-to-do people can pay from thirty shillings to two guineas a week for a nurse, but struggling folk can sometimes barely manage half those sums.

*Nervous Disorders.*

(8) I should be glad to know if any doctors now advocate the removal of the ovaries in nervous cases. I understand that there has been discussion lately on the subject in medical papers.—*H. S.*

There is a strong opinion, which is gaining ground, that healthy ovaries should not be subjected to surgical interference. The contrary view has been held by a small section of surgeons, but the operations have been more or less of an experimental nature.

*Royal Red Cross.*

(9) Can you tell me under what conditions the Royal Red Cross may be won? Is it only given for work connected with the army?—*F. R.*

The Royal Red Cross is a decoration for women, instituted in 1883 for rewarding devotion in providing for, and nursing, sick and wounded soldiers, sailors and others with the Army in the field, on board ship, or in hospitals.

*Medical Training.*

(10) Will you kindly tell me in this week's Hospital how a woman may study to become a doctor?—*M. I. N. H.*

A great number of our readers wish to have their answers inserted in the Hospital of the week following their letter. This is quite impossible in consequence of the very large number of queries received. A fee of half-a-crown enclosed would enable them to have an immediate reply privately. Write to the Secretary of the London (Royal Free Hospital) School of Medicine for Women, 8 Hunter Street, Brunswick Square, W.C., for particulars.

*Hospital Training.*

(11) I am 22 years of age, and would like to go in for general training. Do you think one year in fever training long enough, or is it better to have a three-years' certificate?—*J. H. H.*

You must have a three-years' certificate to be a general nurse; after that a year's training in fever nursing would be useful. See replies to our other correspondents under this heading.

**Standard Nursing Manuals.**

"The Nursing Profession: How and Where to Train." 2s. net; post free 2s. 4d.
"A Handbook for Nurses." (Illustrated). 5s.
"Nursing: Its Theory and Practice." (New Edition.) 3s. 6d.
"Nursing in Diseases of Throat, Nose, and Ear." 2s. 6d.
"Surgical Ward Work and Nursing." (Revised Edition.) 3s. 6d. net; post free, 3s. 10d.
"Art of Massage." (Second Edition.) 6s.
"Elementary Physiology for Nurses." 2s.
"Elementary Anatomy and Surgery for Nurses." 2s. 6d.
"Practical Handbook of Midwifery." 6s.
"Mental Nursing." 1s.
"Art of Feeding the Invalid." 1s. 6d.

**All these are published by the Scientific Press, Ltd., and may be obtained through any bookseller or direct from the publisher 28 and 29 Southampton Street, London, W.C.**

